# Remembering a Plastic Surgery Legend: Stanley Klatsky, MD

**DOI:** 10.1093/asjof/ojaa047

**Published:** 2020-11-17

**Authors:** Jeffrey M Kenkel

**Affiliations:** Department of Plastic Surgery, University of Texas Southwestern Medical Center, Dallas, TX

**Figure 1.**
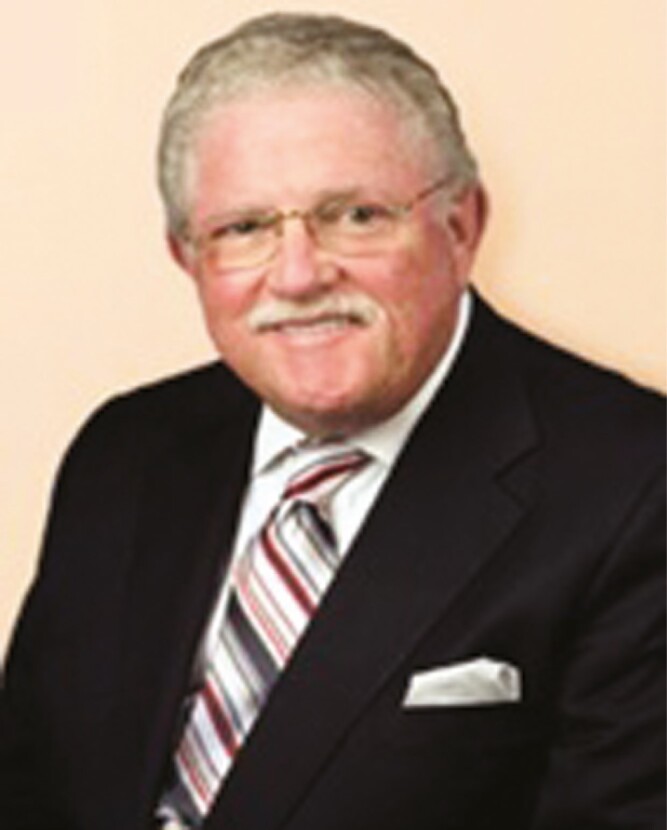
We remember *Aesthetic Surgery Journal* Editor Emeritus, Dr. Stanley Klatsky (1934–2020) through tributes by his peers and past presidents of The Aesthetic Society.

All of us who knew Dr Stanley Klatsky (Figure 1) within the plastic surgery community or served as editors beside him on the *Aesthetic Surgery Journal* (*ASJ*) have lost a legend in our field. His passing has taken us all by surprise; however, he lived his 86 years to the fullest extent with his loving wife Rosalie and his 2 sons Mark and Alan by his side. We will miss his gentle mannerisms, countless soft-spoken conversations, and the wealth of knowledge he shared with us all so selflessly.

In Video 1, we remember and pay tribute to his myriad contributions to plastic surgery, his friendship and mentorship, and more achievements than can be counted. He educated countless plastic surgeons, many of whom now practice and lecture among the leaders in our community. Stan’s wisdom, technical skill, and attention to detail made him a living legend who was among the most revered members of The Aesthetic Society. He only missed one of our Society’s annual meetings in all his years of practice—a true testimony to his love of and commitment to the organization. He will be remembered fondly as Editor Emeritus of *ASJ* who took a fledgling newsletter and transformed it into a leading authority on aesthetic surgery, working strategically behind the scenes to hand off a journal ready for indexing to his successor, Dr Foad Nahai, whom I have worked beside as Associate Editor for the past 10 years. We will never forget his devotion and leadership, and above all, his loyalty to his craft.

Godspeed, Stan—you left the field of plastic surgery so much better than you found it many years ago and we are all the beneficiaries of your talent and goodwill.

